# Defining an approach to empower clinical geneticists to do genomic reanalysis

**DOI:** 10.1186/s12920-025-02293-3

**Published:** 2026-01-05

**Authors:** Michael M. Segal, Meriel McEntagart, Alexander T. Deng, Andrea Haworth, Brian King, Anthony Rogers, John Filby, John Short, Mary Grace Hash, Lynette C. Rives, Kimberly M. Ezell, Undiagnosed Diseases Network, John A Phillips

**Affiliations:** 1 SimulConsult Inc, Chestnut Hill, MA United States; 2 St George’s University NHS Foundation Trust, London, UK; 3 Guy’s & St Thomas’s NHS Foundation Trust, London, UK; 4 Congenica, Cambridge, UK; 5 Vanderbilt University Medical Center, Nashville, TN United States

**Keywords:** Bioinformatics, Clinical exome, Diagnostic yield, Phenotype; reevaluation, NGS-based resequencing, Systematic reanalysis, Clinical genomics, Re-analysis, Sequence analysis

## Abstract

**Background:**

Sequencing reanalysis can benefit from the inclusion of new information about the patient and from the literature. We studied approaches needed to make reanalysis part of routine follow-up by clinical geneticists.

**Methods:**

Reanalysis used the SimulConsult diagnostic decision support software, which generates a pertinence metric for gene zygosities determined from the variant table and the patient’s findings. Twenty patients had routine exome sequencing at St. George’s Hospital (London, UK). Twenty were admitted to the Undiagnosed Diseases Network at Vanderbilt University Medical Center (VUMC) and had all remained undiagnosed despite previous evaluations and sequencing.

**Results:**

For St. George’s cases, reanalysis picked 7 of the 7 initial diagnoses plus 2 diagnoses found later, and suggested another diagnosis with a gene absent from the variant table. For VUMC, reanalysis picked 5 of 8 diagnoses that were in the variant tables, and suggested a non-coding variant absent from the variant table.

**Conclusion:**

Rapid reanalysis by clinicians could increase the yield of genetic diagnosis with minimal effort and no new lab expenses. For the routine cases at St. George’s, diagnostic yield increased from 7 to 10 (43%). Capabilities that could further increase yield include joint variant calling, robust phenotyping, clinical correlation after sequencing, and adding CNV data to variant tables.

## Introduction

Many patients who have undergone genomic sequencing but still lack a diagnosis may be diagnosed in the future as new genetic disorders and phenotypic expansions are published. The best care for such patients is to reassess them for a possible diagnosis periodically, ideally as part of routine follow-up care by clinical geneticists. Such reassessment brings in 4 types of new knowledge:

1. Novel gene-disease associations
2. Disease phenotype expansion in a previously known disease
3. Additional pertinent clinical and lab findings determined to be present or absent in the patient
4. New information about pathogenicity of specific variants in a gene

A full commercial genomic reanalysis that reflects these 4 types of new information can be very labor intensive and costly [1–6] and require submission of new clinical information to the lab. However, with advances in software for clinical correlation, a broader reanalysis could be made more accessible so that it could be done by clinical geneticists. This can empower the clinicians who follow the patient to make maximal use of updated findings as well as knowledge from the literature [7, 8]. We studied the practicality of making such reanalysis part of the routine follow-up care by the clinician best positioned to do the clinical correlation: the patient’s geneticist, and the capabilities needed to make the reanalysis straightforward enough to be done routinely by clinical geneticists.

This work was presented in preliminary form [9].

## Materials and methodology

We used two cohorts of patients, 20 from each site. The first cohort received regular clinical exome testing at St. George’s Hospital NHS Foundation Trust in the UK and diagnoses had been found in 7. The second cohort was from the Undiagnosed Diseases Network (UDN) clinical site at Vanderbilt University Medical Center (VUMC) in Nashville TN, whose cases were more challenging and all had been undiagnosed after initial assessment and sequencing.

Annotated variant table files were generated by the digital health company Congenica. For the UK cases, Congenica had the underlying Binary Alignment/Map (BAM) files from which it generated VCF files to annotate, but for the VUMC cases Congenica only had the Variant Call Format (VCF) files, prepared using VUMS's own analysis pipeline using the BAM files from sequencing done by the UDN sequencing core labs. However, VUMC had the BAM files and was able to consult them manually in assessing plausibility of variants. Although the annotated variant tables used by the software can include copy number variation (CNV) for chromosomal regions [10], neither of the sites here was equipped to do this procedure so such information was not included in the variant tables used in this study.

All Next Generation Sequencing (NGS) findings from the pilot study were confirmed by Sanger sequencing and reports were issued from the diagnostic laboratory. At both sites there were existing provisions for informing families of changes in the analysis.

For the results reported here, variants were ignored if their frequency was greater than the frequency expected from the incidence of the corresponding diseases. The analysis was also checked without this filtering and no diagnoses were changed.

The reanalysis was done using the Genome-Phenome version of the SimulConsult diagnostic decision support software [10]. The software uses patient clinical and laboratory findings input manually into the software or by Natural Language Processing of pasted text, including both pertinent positive findings (with onset) and pertinent negative findings. The clinician loads the annotated variant table file and the software displays in seconds a differential diagnosis and a list of gene zygosities (e.g., biallelic) found in the variant table. These are ranked not by severity but by a metric of pertinence calculated by removing from the analysis in turn each gene zygosity finding ascertained from the variant table, recalculating the differential diagnosis, and comparing how different the differential diagnosis would have been without that variant [10] and is described in detail [11]. The pertinence score has been shown to be highly suggestive of the correct diagnosis [12].

The analysis compares these phenotypic elements to knowledge in a large, curated database that includes frequency and onset of individual findings in diseases, allowing detailed comparisons with known phenotype and the use of pertinent negative findings in genomic diagnosis. It includes > 9,600 diseases, both genetic and nongenetic, and the > 4,800 genes with germline changes convincingly associated with human disease. It also includes chromosomal syndromes. It does so with > 260,000 associations of findings with diseases curated from the literature by clinicians.

Clinical descriptions were provided at each site. At St. George’s these included all findings considered relevant to the sought disease. At VUMC a subset of such findings were chosen by the clinical, molecular and research geneticists and structural biologists. At St. George’s, after importing and analyzing the variant table, medical records were checked in a clinical correlation step [13] to comment on additional findings suggested by the software as being useful based on the abnormal variants found; these were typically pertinent negative findings. At VUMC such a step was not included.

## Results

For the St George’s cases, where Congenica had the underlying BAM files with which to prepare the VCF files, the number of de novo variant calls in the annotated variant tables of trios averaged 71. For the VUMC cases, where Congenica only had the VCF files, the number of de novo variants for trios averaged 406. Correspondingly, the average number of possible gene zygosities with known clinical phenotype, whether they fit the patient phenotype or not, was 18.6 for the St. George’s cases and 31.4 for VUMC cases.

### St. George’s cases

There were 20 St. George’s cases; 18 trios and 2 cases with only one parent (Table 1). In 7 cases, diagnoses with genes in the variant table were called in the original analysis, and all 7 were ranked #1 in the software. One more that was ranked #1 (*TAB2*) was established later by St George’s by correlation with a case reported in Wade et al. [14] and another (*PAX3*) was uncovered by St. George’s after further testing. Thus, of the 20 diagnoses there were 9 cases with diagnoses with genes that had variants in the original variant table, though only 7 were called originally. All 9 cases with a Congenica/St. George’s genomic diagnosis were ranked as the #1 zygosity in the software, including the 2 diagnoses not made until later (Table 1).

**Table 1:** St. George’s cases

**Case**	**Sex**	**Status of parents**	**Gold standard gene**	**Simul Consult rank of gold standard gene**	**Simul Consult #1 gene**	**Zygosity**	***De novo***	**Simul Consult disease rank**	**Pertinence**	***De novo*** ** #**	**Number of zygosities listed**
1	M	2 OK	WDR26	1	WDR26	monoallelic	Y	1	100.000	73	12
2	M	2 OK	ARID1B	1	ARID1B	monoallelic	Y	1	100.000	83	12
3	M	♀ OK	OCRL	1	OCRL	X-linked	N	1	62.130	N/A	54
4	M	2 OK	KANSL1	1	KANSL1	monoallelic	Y	1	100.000	90	22
5	M	2 OK	PURA	1	PURA	monoallelic	Y	1	100.000	43	17
6	M	2 OK	PAX3	1	PAX3	monoallelic	Y	71	0.003	52	10
7	F	2 OK	EIF2B5	1	EIF2B5	biallelic	N	36	0.000	39	8
8	M	2 OK	?		ALG13			183	0.000	72	12
9	M	2 OK	TAB2	1	TAB2	monoallelic	Y	155	0.000	72	11
10	M	2 OK	?		AR			10	1.598	73	13
11	M	2 OK	?		NCP1			120	0.000	41	13
12	M	2 OK	?		SHANK3			1	100.000	74	17
13	F	2 OK	CAPN3	N/A	GAA	deletion	N	9	0.023	226	20
14	F	2 OK	?		CHMP1A			1	1.823	34	14
15	F	2 OK	?		PIBF1			26	0.122	70	11
16	M	2 OK	?		PDHA1			414	0.000	49	7
17	M	2 OK	?		APOB			63	0.003	47	18
18	F	♂ affected	?		COL11A1			8	3.393	N/A	70
19	F	2 OK	?		FAM20C			73	0.000	88	16
20	M	2 OK	SLC5A6	1	SLC5A6	biallelic	N	1	100.000	45	15

Status of parents is listed as 2 OK if both were unaffected; in cases 3 and 18 only one parent could be assessed. Gold Standard gene is the gene determined to be causative if such a determination could be made, and zygosities are included in these instances. SimulConsult rank is the rank of the gene in the SimulConsult software, while SimulConsult #1 gene refers to the gene zygosity ranked #1 in the software. Pertinence is the metric described previously [10-12]. De novo # refers to the number of de novo variants. Number of zygosities listed refers to the number of abnormal gene zygosities in the variant table

**Fig. 1:**
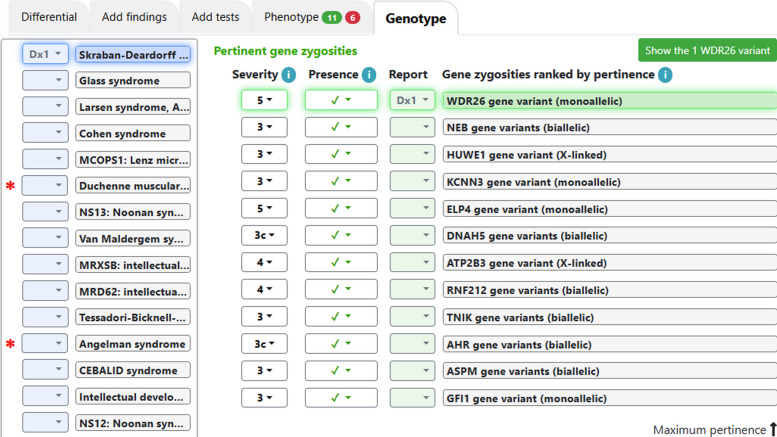
*WDR26* has 100% pertinence, suggesting the diagnosis of Skraban-Deardorff syndrome. Legend: On the left are diseases in the differential diagnosis after importing the variant table. Disease probability is shown in blue shading. On the right are all the abnormal gene zygosities with significant severity (on a scale from 0 to 5) deduced from the variant table, ranked by pertinence in having affected the differential diagnosis [10, 11], not by severity. Only *WDR26* fit a disease high in the differential diagnosis; the next gene, *NEB*, fit the disease #61 in the differential diagnosis. The clinician using the software has labeled the disease and the gene as the diagnosis.

For each of the gene zygosities known to be associated with clinically described diseases, the pertinence metric for genes was displayed as a green bar over the gene zygosity (Fig. 1); the numeric values are included in Table 1 as a percentage of the maximum pertinence for all findings (including clinical, gene zygosities and other lab results). In 5 cases the pertinence metric was 100% (e.g., Fig. 1); the average pertinence for the #1 zygosity for these 9 cases was 62.5%. For the 11 cases with no diagnosis there was (by definition) a top zygosity for each case, but the average pertinence was 9.7%, with only 1 of 11 of those cases having scores over 4%. The top zygosity in that case (*SHANK3*) was dismissed as the diagnosis because of low read numbers and apparent biallelic zygosity despite parents not having the variant.

For this study there was no contact with any of the patients as part of the reanalysis, but in 7 cases there was a clinical correlation step after importing the variant table, consisting of additional chart review and examination of photos in the chart to comment on findings ranked as Useful in the Add findings and Add Tests tab of the software. Changes were made for 7 cases, typically pertinent negative findings. In 2 cases there was no change in the 100% pertinence metric (*KANSL1* and *SLC5A6*). In 3 cases there was only a minor increase in low pertinence (*PAX3*, *TAB2* and *EIF2B5*), but in the *PAX3* case the diagnosis was considered correct because the patient had dystopia canthorum, which was judged by the geneticists to be very specific but was bundled in the software together with telecanthus, which is less specific. In one case pertinence decreased from 100.0% to the 62.1% shown in Table 1, arguing that the diagnosis was atypical, but not enough to change the diagnosis. In one case that remains undiagnosed, addition of negative Magnetic Resonance Imaging (MRI) findings reduced pertinence of *CHMP1A* from 60.1% to 1.8%, making that possibility seem unlikely (Case 14).

**Fig. 2:**
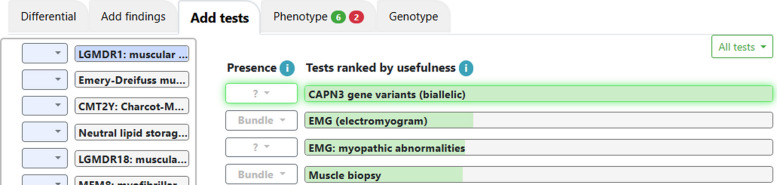
On the left are diseases in the differential diagnosis after importing the variant table. Disease probability is shown in blue shading, and Limb-girdle Muscular Dystrophy type 1 is ranked highest. *CAPN3* is recommended as most useful test, with green shading representing a metric of usefulness for changing the differential diagnosis [11], despite no variants in the gene having been in the variant table.

For one case, following the importation of the variant table, the top suggestion in the Add Tests tab was to check for a variant in *CAPN3* (Fig. 2; green shading), which did not appear in the variant table. *CAPN3* was ranked high based on the strong clinical match with the disease autosomal recessive limb-girdle muscular dystrophy-1 (LGMDR1; Fig. 2; blue shading). Subsequent copy number testing revealed a 2-exon deletion in *CAPN3* variants, which was deemed to be diagnostic.

Abnormalities were de novo in 6/10 (60%) diagnosed St. George’s cases, all monoallelic.

### VUMC Cases

There were 20 VUMC cases, all of which were all undiagnosed after initial assessment and sequencing and then referred to the UDN. In the UDN analysis, 10 were diagnosed and 10 were not despite extensive evaluations (Table 2). These 20 cases included 16 trios, and 4 cases with only a proband and an unaffected mother. For the VUMC cases only a subset of the known findings chosen by VUMC was used in the analysis and there was no step of clinical correlation after importing the variant table. Of the 10 diagnosed cases, only 8 had convincing variants in the variant table. Two more were diagnosed based on copy number analysis (*STXBP1* and *SSR4*); although there were variants in these 2 genes in the variant table, they were unconvincing.

**Table 2:** VUMC cases

**Case**	**Sex**	**Status of parents**	**Gold standard gene**	**Simul Consult rank of gold standard gene**	**Simul Consult #1 gene**	**Zygosity**	***De novo***	**Simul Consult disease rank**	**Pertinence**	***De novo*** ** #**	**Number of zygosities listed**
1	F	♀ OK	GRIN2B	16	ATN1	monoallelic	N/A	14	0.126	N/A	46
2	F	2 OK	?		PIBF1			127	0.000	162	18
3	F	2 OK	?		PDHA1			71	0.000	150	21
4	M	2 OK	AMER1	1	AMER1	X-linked	Y	6	2.315	169	16
5	F	♀ OK	SELENON	1	SELENON	biallelic	N/A	1	100.000	N/A	63
6	M	2 OK	FBXO11	1	FBXO11	monoallelic	Y	1	100.000	151	15
7	M	2 OK	RNU4-2	N/A	CREBBP	monoallelic	Y	46	0.026	249	32
8	F	2 OK	EFTUD2	1	EFTUD2	monoallelic	Y	1	100.000	94	11
9	M	2 OK	?		ETFA			1728	0.000	223	25
10	F	2 OK	?		CNOT1			5	0.440	183	26
11	M	2 OK	?		DEAF1			24	0.000	918	84
12	F	2 OK	?		DEAF1			11	0.334	166	21
13	F	2 OK	FA2H	1	FA2H	biallelic	N	18	0.000	92	17
14	F	♀ OK	?		EHMT1			1	96.603	N/A	60
15	F	♀ affected	NR2F2	6	KDM6B	monoallelic	N	78	0.000	1051	15
16	F	2 OK	STXBP1	N/A	TCF4	deletion	Y	13	0.257	1309	22
17	M	2 OK	OPHN1	3	ATN1	X-linked	Y	14	0.063	82	20
18	F	2 OK	?		MTMR14			455	0.000	89	16
19	M	2 OK	?		TPP1			23	0.000	1406	21
20	M	♀ OK	SSR4	N/A	CNOT1	deletion	N/A	1	87.408	N/A	78

Status of parents is listed as 2 OK if both were unaffected; in cases 5, 15 and 20 only one parent could be assessed, and zygosities are included in these instances. Gold Standard gene is the gene determined to be causative if such a determination could be made. SimulConsult rank is the rank of the gene in the SimulConsult software, while SimulConsult #1 gene refers to the gene zygosity ranked #1 in the software. Pertinence is the metric described previously [10-12]. De novo # refers to the number of de novo variants. Number of zygosities listed refers to the number of abnormal gene zygosities in the variant table

For the remaining 8 cases, the causative variants were ranked #1 in 5 of the cases (Table 2). In 3 of these cases the pertinence metric was 100%; the average pertinence for the top zygosity for these 5 cases was 60.5%. Of the remaining 3 cases in which the diagnosis was not #1, the following was found:

- *GRIN2B*: The gene was initially #16 in pertinence. The diagnosis was made by VUMC based on 5 cases in the literature with the same monoallelic variant.
- *NR2F2*: The gene was initially #6 in pertinence. The diagnosis was made by VUMC based on the partially affected mother being mosaic for the variant.
- *OPHN1*: The gene was initially #3 in pertinence. This would have been #1 if 3 noncurated findings in the Online Mendelian Inheritance in Man (**OMIM**) Clinical Synopsis had been curated into the SimulConsult database.

To better compare the VUMC cases to the St. George’s cases, a step of clinical correlation by chart review was simulated for 2 of the VUMC cases. This was done by adding pertinent positives used in VUMC’s internal analysis but not submitted for the software analysis, and by adding some pertinent negatives from chart review. For *GRIN2B* the gene pertinence went from #16 to #11. For *OPHN1* the gene pertinence rose from #3 to #2.

In one undiagnosed case, the ultimate diagnosis was *RNU4-2*, a non-coding gene which did not appear in the original variant table. However, an 88-center study including VUMC [15] defined ReNU syndrome and Sanger sequencing revealed a de novo variant in the proband. The disease had been added a part of routine curation of new data in the published literature into the SimulConsult database and *RNU4-2* was listed as #1 in usefulness of further genetic testing based on the strong clinical match with ReNU syndrome, similar to that for *CAPN3* in Fig. 2.

For the other 9 cases undiagnosed by VUMC, only one had a pertinence score over 0.5 (case 14), but the read depth for *EHMT1* in the BAM file was too low and the variant appeared to be in an untranslated region.

Abnormalities were de novo in 5/7 (71%) of the diagnosed cases in which that could be determined; in 3 of these 11 diagnosed cases the father’s DNA was not sequenced.

## Discussion

This study assessed the feasibility and potential use of software-based genomic reanalysis and the factors that can improve the diagnostic rate.

The diagnostic rate was best in the St. George’s cases, in which:

- the cases were more routine
- no patients were excluded from the study on the basis of prior sequencing, versus the VUMC UDN patients where prior sequencing filtered out straightforward cases
- the number of de novo variants was lower because of access to the BAM files
- there was clinical correlation after inclusion of the variant table data

For the St. George’s cases the number of diagnoses was boosted from 7 to 9, and if one includes the suggested *CAPN3* testing, boosted to 10, an increase of 43% in diagnostic rate.

For the VUMC cases the reanalysis was more challenging because all the cases previously had multiple evaluations with no diagnosis found before UDN acceptance, often including panel, exome or genome sequencing. In addition, there were excessive numbers of false de novo calls because of lack of joint variant calling. One improvement was suggesting *RNU4-2* analysis after subsequent discovery of ReNU syndrome. Diagnostic accuracy would have improved with clinical correlation after inclusion of the variant table data.

### Limitations

The purpose of this study was to define an approach to empowering clinical geneticists to do genomic reanalysis that would be feasible in a variety of clinical genetics situations. Although two sites were used to surface different issues in different settings, it was not a goal to do a statistical comparison of results at different sites according to difference in protocols at those sites and the numbers of cases were not scaled up to do such comparisons. Permission was not requested for the international transfer of VUMC BAM files that would have been needed to control for differences in generating VCF files. The differences in the difficulty of cases and the process for generating VCF files are mentioned in order to explain the non-surprising differences in diagnostic rates and de novo variants.

Although the analysis software was equipped to analyze variant tables with CNV information as we have done previously [10], neither of the sites here was equipped to generate this data. Although we have shown that the analysis is greatly sharpened by using joint variant calling [12], neither of the sites here was equipped to do this procedure.

## Conclusions

The results suggest that there is utility to clinical geneticists being able to do an informal genomic reanalysis, triggered by either new clinical information or potential new gene-disease discoveries or expansion of existing phenotypes [16]. This was evident both for standard cases and for the VUMC cases, which were more difficult unsolved cases submitted to a UDN site.

The results suggest a roadmap of 7 capabilities needed to make reanalysis straightforward enough to be done routinely by clinical geneticists:

1. *Joint variant calling and BAM-aware methods*: The totals of de novo variants of 71 at St. George’s and 406 at VUMC are far above the number of true de novo variants. In previous work we were able to reduce the numbers to an average of 3.2 using BAM-aware joint variant calling [12]. Reducing the number of false de novo calls is crucial because in 11 of 17 (65%) of the new reanalysis diagnoses here, the variant identified as pathogenic was de novo. Access to the BAM files for the VUMC cases would undoubtably help identify false de novo calls. Variants called in the proband may be falsely determined as de novo due several reasons: mosaicism in the parent, low coverage in the parent leading to no call, or low coverage in the proband leading to variant calling with low confidence. The presence of reads supporting a call in both the proband and a parent is much stronger evidence for the existence of a real variant and having access to the BAM files allows for the identification of evidence below the threshold for calling in the individual.
2. *Robust initial phenotype*: Using a wide set of phenotypic findings, particularly pertinent negative findings to help prioritize diagnoses.
3. *Clinical correlation after genomic testing*: Further findings can be added after importing the variants or during follow-up visits, guided by the software’s usefulness metric, which takes into account the gene variants. Furthermore, as demonstrated in 2 of the cases (CAPN3 and RNU4-2), clinical correlation suggested testing genes missing from the variant tables that proved to be pathogenic.
4. *CNV data*: Incorporating CNV regions in the variant table as we have done previously [10] would likely have found the 2 CNV diagnoses at VUMC (STXBP1 and SSR4) and one at St. George’s (CAPN3).
5. *Non-coding variants*: Non-coding variants and other new types of variants that are discovered but not included in current sequencing pipelines.
6. *Case-based quality improvement*: In one of the cases (OPHN1), the pathogenic gene would have risen to #1 if the person analyzing the genomic data had curator privileges and could have added 3 findings in the OMIM Clinical Synopsis that were absent from the curation for the genetic condition that was pathogenic.
7. *Known pathogenicity annotation*: Using the field in the annotated variant table for information about known pathogenicity of particular variants from efforts such as ClinVar would be helpful, as illustrated by the GRIN2B case.

All 7 capabilities are used in formal laboratory analysis, but to make reanalysis more routine all 7 could be accomplished in a straightforward manner using current technology, and only #6, the pathogenicity annotation, would require generating a new variant table. For patients who remain undiagnosed, a process as described here could allow a clinical geneticist to do a reanalysis of genomic data in minutes as part of routine follow-up appointments, only needing to request a new lab-based analysis when a new diagnosis appears likely. The data presented here suggest that an increase in diagnostic yield could be as high as the 43% noted in the St. George’s cohort data.

## Data Availability

The datasets used and/or analyzed during the current study are available from the corresponding author on reasonable request. Genes but not DNA sequences or variants are named in this study. As this study involved the reanalysis of pre-existing sequencing and variant data no new or novel sequence or variant data has been deposited into accessible databases. For the St. George’s data all findings were Sanger confirmed and reported by the UKAS accredited diagnostic service. For the VUMC patients, the datasets generated and/or analyzed during the current study are available in the UDN repository, (https://gateway.undiagnosed.hms.harvard.edu).

## Abbreviations

BAM: Binary alignment/map
IRB: Institutional review board
MRI: Magnetic resonance imaging
NGS: Next generation sequencing
OMIM: Online Mendelian inheritance in man
UDN: Undiagnosed diseases network
VCF: Variant call format
VUMC: Vanderbilt university medical center
